# Gestational Exposure to Silymarin Increases Susceptibility of BALB/c Mice Fetuses to Apoptosis

**Published:** 2017

**Authors:** Mahbobe Gholami, Seyed Adel Moallem, Mohammad Afshar, Leila Etemad, Gholamreza Karimi

**Affiliations:** 1. Department of Pharmacodynamics and Toxicology, Faculty of Pharmacy, Mashhad University of Medical Sciences, Mashhad, Iran; 2. Pharmaceutical Research Center, Mashhad University of Medical Sciences, Mashhad, Iran; 3. Department of Anatomy, Birjand University of Medical Sciences, Birjand, Iran; 4. Pharmaceutical Research Center, Faculty of Pharmacy, Mashhad University of Medical Sciences, Mashhad, Iran; 5. Department of Nursing, Faculty of Midwifery, Neyshabur University of Medical Sciences, Neyshabur, Iran

**Keywords:** Apoptosis, Fetus, Silymarin, Teratogenicity

## Abstract

**Background:**

Silymarin is a flavonolignan that has been the subject of research to evaluate the beneficial properties for decades. Silymarin has been known for its potent cytoprotective, hepatoprotective and antioxidant activities. The goal of the present study was to gain a deeper understanding of possible molecular mechanisms of apoptosis of the injuries induced by silymarin on BALB/c mice fetuses.

**Methods:**

The present experimental study was carried out in virgin female BALB/c mice. The animals were divided randomly into 4 groups. Three test groups were injected intraperitoneally with silymarin at doses of 50, 100 and 200 *mg/kg/day* during gestational days 6–15. The control group received the solvent by the same route at equivalent volume. Western blot analysis was conducted to determine the levels of caspase-3 and caspase-8 in fetal heart, kidney, lungs and brain tissue.

**Results:**

The results of this study showed that silymarin administration during organogenesis at doses of 50, 100 and 200 *mg/kg* can significantly increase the protein levels of caspase-3 and 8 in heart, kidneys and brain tissues of mice fetuses compared with control group (p<0.001). Silymarin exposure could not change the level of apoptotic markers in fetal lung tissue.

**Conclusion:**

According to the results, programmed cell death, especially via the intrinsic pathway, plays a pivotal role in the pathogenesis of silymarin-induced malformations in some tissue including heart, kidneys and brain. More studies are needed to determine other molecular mechanisms underlying silymarin- induced embryo toxicity.

## Introduction

Silymarin is a flavonolignan that is ^1,2^ extracted from the seeds and fruit of *Silybum marianum* (milk thistle) plant. This flavonoid complex has been the subject of research to evaluate the beneficial properties for decades. Silymarin contains several flavonoids most notably, silybinin (or silybin as the most active fraction), silydianine, and silychristine ^2^. Silymarin has been known for its potent cytoprotective and hepatoprotective activities ^3^. Silymarin can treat various liver disorders and regenerate the liver cells ^3^. Silymarin and silybinin, due to their antioxidant action, have the capacity to prevent the progress of various neurodegenerative processes, cardiopulmonary and gastrointestinal problems and liver damages. There is strong evidence in *in vitro* and *in vivo* studies that silymarin interferes with promotion and progression of cancer ^4^. Silymarin possesses anti oxidant activity by acting as a free radical scavenger influencing the enzymatic system associated with GSH and SOD ^5,6^. Beside antioxidant activity, other mechanisms may contribute to the beneficial effect of silymarin. Some of the most important ones are cell membrane stabilizing, stimulation of ribosomal RNA polymerase and subsequent protein synthesis, inhibition of neutrophil migration and cyclin- dependent kinase, immunomodulatory and anti-inflammatory effects ^7^.

Pharmacokinetic studies have shown that silymarin is absorbed when given orally ^8^. During the pregnancy, silymarin can cross the placental barrier and produce measurable amounts in fetal tissue. However, there is not enough information concerning exposure to silymarin during pregnancy ^9^. In our previous research, it was found that silymarin administration during organogenesis in mice can cause fetal resorption, intrauterine growth retardation and skeletal malformations ^10^. It was also found that some brain lesions are associated with apoptosis and oedema. Indeed, cardiac congestion and immaturity and infiltration of inflammatory cells in lungs and kidneys were observed ^11^. Programmed cell death or apoptosis is a crucial process during embryonic development. Defective apoptosis can result in abnormal development and pathogenesis.

It involves a cascade of signal transduction steps resulting in the activation of a number of cysteine proteases known as caspases. The goal of the present study was to gain a deeper understanding of possible molecular mechanisms of apoptosis behind the injuries induced by silymarin in BALB/c mice fetuses.

## Materials and Methods

### Animal and treatment

The present experimental study was carried out on virgin female BALB/c mice weighing 20–30 *g* at approximately 2 months of age. They were obtained from Avicenna Research Institute at Mashhad University of Medical Sciences. The mice were kept in 12 *hr* light/dark cycles at a room temperature of 23±2°*C* with unlimited access to food and water. All animal experiments were approved by the Animal Care and Ethics Committee of Mashhad University of Medical Sciences.

One male was caged with two females overnight and each female was examined for the presence of a vaginal plug the next morning. The presence of a vaginal plug was considered as Gestational Day zero (GD0).

Silymarin was purchased from sigma Aldrich Inc. (St. Louis, USA). The mice were randomly divided into four groups. Three groups of pregnant mice were intraperitoneally (IP) injected with silymarin at doses of 50, 100 and 200 *mg/kg/d* during gestational days 6–15 (organogenesis period). Normal saline with a few drops of tween 80 was used as the solvent. The control group received normal saline plus tween by the same route at equivalent volumes.

On GD18, the pregnant mice were sacrificed under chloroform anesthesia and cesarean sections were performed. The fetuses were removed from the uterus. After cutting the umbilical cord, a horizontal incision was made in the neck and a vertical incision in the skulls of the embryos and brains were removed. By making a ventral midline incision in the abdomen, other vital organs including kidneys, heart and lungs were also dissected. Samples were immediately frozen in liquid nitrogen and kept at −80°*C* in a freezer. Six embryos were selected randomly from each litter.

### Western blot analysis

Western blot analysis was conducted to determine the levels of caspase-3 and caspase-8 in fetal heart, kidneys, lungs and brain tissue.

As was explained before, the tissue samples were homogenized in lysis buffer containing Tris 50 *mM* pH=7.4, 2 *mM* Ethylene Diamine Tetra Acetic acid (EDTA), 10 *mM* NaF, 1 *mM* sodium orthovanadate (Na_3_VO_4_), 10 *mM* b-glycerol-phosphate, 0.2% W/V sodium deoxycholate, 1 *mM* phenyl methyl sulfonyl fluoride and complete protease inhibitor cocktail in ice and then were centrifuged at 10,000 *g* for 30 *s* at 4°*C*. Protein concentrations of the samples were measured by Bradford protein assay kit. The protein extracts were then resolved by SDS-buffer and boiled for 5 *min*. The total proteins were electrophoresed in SDS-PAGE gel and were transferred to PVDF membrane as described previously. The blots were probed with caspase-3 (cell signaling #9665) and caspase-8 (cell signaling #4790) primary antibody in 1000-fold dilution for 2 *hr* at room temperature. After washing, they were incubated with secondary antibody (#7074 Cell Signaling). Optical densities of bands were detected with Alliance gel doc (Alliance 4.7 Gel doc, UK) using Enhanced Chemiluminescence (ECL) reagent. The protein bands were analyzed using UVtec software and were normalized against β-actin intensity ^12^.

### Statistical analysis

All data was expressed as mean±SEM and statistical significance was evaluated by one-way analysis of variance (ANOVA) followed by Tukey-Kramer test. Data analysis was done using SPSS version 16.0 software. Differences were considered significant at p-value less than 0.05.

## Results

**Figure 1.**
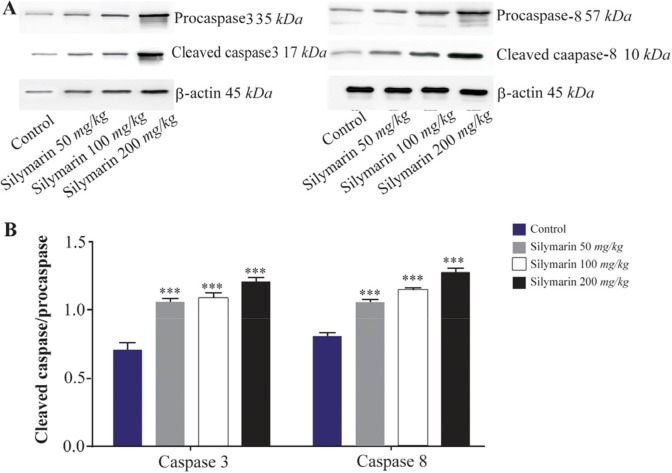
Effect of silymarin on the protein level of caspase-3 and 8 in heart of mice fetuses. The animal groups received 0, 50, 100 and 200 *mg/kg/day* of silymarin. A) Representative photograph for western blot of caspase-3 and 8. B) Densitometric data of protein analysis. Data are expressed as mean±SEM. *p<0.05 compared to the control group.

**Figure 2.**
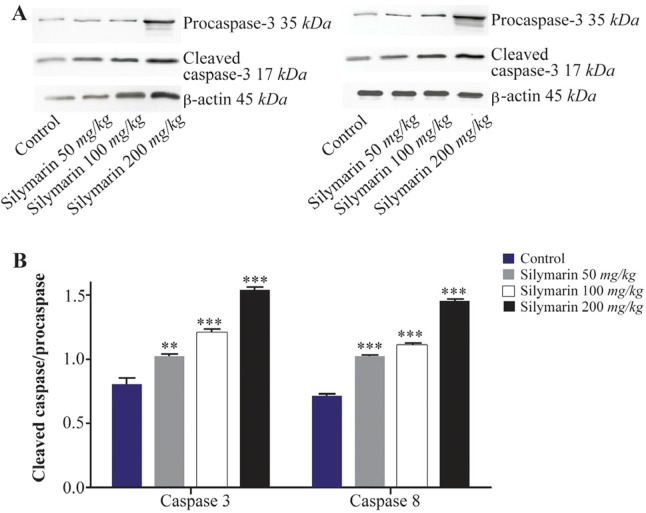
Effect of silymarin on the protein level of caspase-3 and 8 in kidneys of mice fetuses. The animal groups received 0, 50, 100 and 200 *mg/kg/day* of silymarin. A) Representative image showing a Western blot of caspases-3 and 8. B) Densitometric data of protein analysis. Data are expressed as mean±SEM. *p<0.001 compared to the control group.

**Figure 3.**
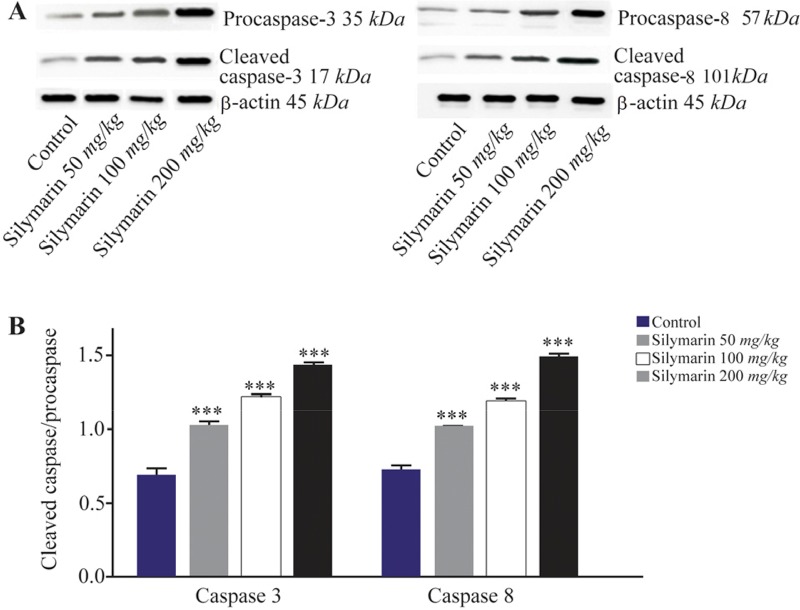
Effect of silymarin on the protein level of caspase-3 and 8 in brain of mice fetuses. The animal groups received 0, 50, 100 and 200 *mg/kg/day* of silymarin. A) Representative photograph of a western blot analysis of the two markers ofcaspase 3 and 8. B) Densitometric data of protein analysis. Data are expressed as mean±SEM. ***p<0.001 compared to the control group.

**Figure 4.**
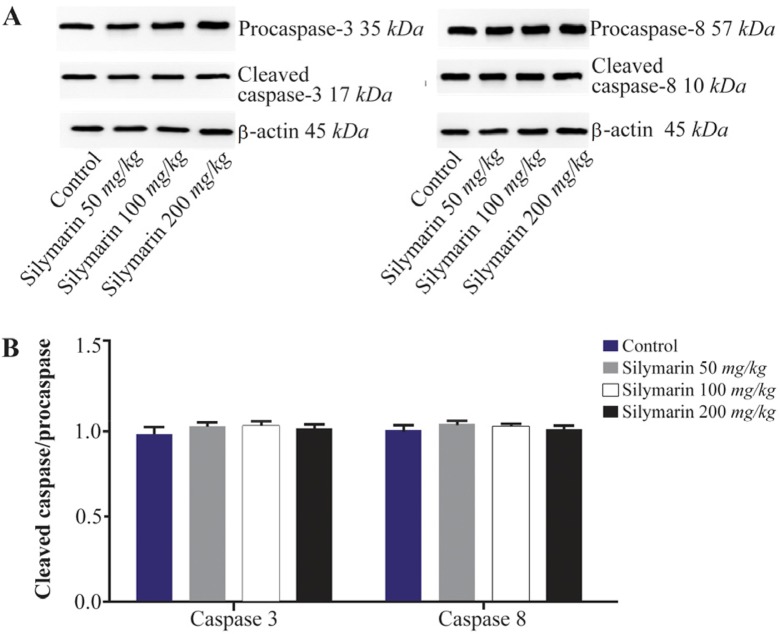
Effect of silymarin on the protein level of caspase-3 and 8 in lungs of mice fetuses. The animal groups received 0, 50, 100 and 200 *mg/kg/day* of silymarin. A) Representative photograph for Western blot of caspase 3 and 8. B) Densitometric data of protein analysis. Data are expressed as mean±SEM.

Expressions of caspase-3 and 8 were measured in different fetal organs including brain, kidneys, heart and lungs. The results expressed in figure 1 showed that silymarin administration during organogenesis at doses of 50, 100 and 200 *mg/kg* can significantly increase the protein levels of caspase-3 in heart of mice fetuses compared with control group (p<0.001). The activation of caspase 8 was also measured as a crucial mediator of the death receptor pathway. The myocardial protein expressions of caspase 8 were up-regulated by silymarin at all treated groups in comparison with control group (p<0.001). The group that received 200 *mg/kg* was significantly higher at caspase 8 level compared with 50 and 100 *mg/kg* groups (p<0.001) (Figure 1). Evaluation of the effect of silymarin on fetal kidneys tissue revealed that silymarin can significantly increase expression of caspase 3 at all doses tested compared with control group (p<0.01). The level of caspase 8 also increased significantly at all treated groups (p<0.001) (Figure 2). The differences between third and other treated groups were also completely significant at protein level of caspase-3 and 8 in renal embryonic tissue (p<0.001). In addition, the brain was affected by silymarin and showed increased expression level of caspase-3 and 8 compared with the control group (p<0.005). There were significant differences between all treated groups (Figure 3). However, the results demonstrated that silymarin cannot change the level of caspase 3 and 8 activity in fetal lung tissue compared with the control group at different doses (p>0.05) (Figure 4).

## Discussion

In our previous study, it was showed that silymarin administration at different doses can cause embryonic resorption, intrauterine growth retardation and some kind of malformations including limb, vertebral column and craniofacial abnormalities ^10^. Pathologic findings in another study also revealed that silymarin administration during organogenesis induces oedema and apoptosis in foetal cells of brain with maximal effect at highest dose. It also showed immaturity and infiltration of inflammatory cells in lungs as well as cardiac congestion ^11^. According to results of this study, administration of silymarin at all doses induces apoptosis in brain, kidneys and heart involving caspase-8 which then activates caspase-3. Active caspase-8 can play an important role in extrinsic apoptosis directly by activating executioner caspase-3 or activates the mitochondrial apoptosis pathway through cleavage of BID ^13^. The intensity of damage was increased in all fetal tissues in a dose-dependent manner. Therefore, extrinsic pathways of apoptosis may be one mechanism contributing to different abnormalities among fetuses with prenatal silymarin exposure.

On the other hand, apoptosis has been introduced as a known mechanism of action of silymarin that provides protection against cancers. There are some reports on the reduction in apoptotic gene/protein expression in different cancer cell lines treated with silymarin ^14–17^. Silymarin treatment resulted in a reduction of cytochrome C release, Bcl-2 protein level and increasing Bax and activation of caspase-3 on human ovarian cancer cell lines ^17^. Silymarin also inhibited population growth of the human hepatocellular carcinoma cell line (HepG2) in a dose-dependent manner and increased the percentage of cell death through mitochondrial apoptotic pathway ^15^. Indeed, evaluation of the effect of silybinin on the prostate carcinoma DU145 cells and bladder transitional-cell papilloma RT4 cells revealed that silybinin can cause caspase-mediated apoptosis by inhibition of stat3 and increasing the level of p53 protein, respectively ^18,19^. Animal studies have also indicated that silymarin may express antiapoptotic properties in renal impairment, cardiomyopathy and neuronal injury ^20–22^.

It seems that apoptosis effect of silymarin depends on biological differences between embryos and adults as well as pathologic condition.

But silymarin administration during organogenesis did not induce significant changes at caspases protein levels in lung tissue and it is concluded that silymarin induced fetal lung injury is not in the least related to caspase dependent apoptosis. However, according to the result from *in vitro* assay, silymarin can induce apoptosis in a lung cancer cell line *via* the mitochondria-dependent caspase cascade pathway ^23^.

## Conclusion

According to the results, programmed cell death, especially via the intrinsic pathway plays a pivotal role in the pathogenesis of silymarin-induced malformations in some tissue including heart, kidneys and brain. More studies are needed to determine other molecular mechanisms underlying silymarin-induced embryo toxicity.
